# Gauging response time distributions to examine the effect of facial expression inversion

**DOI:** 10.3389/fpsyg.2023.957160

**Published:** 2023-02-24

**Authors:** David L. Bimler, Galina V. Paramei

**Affiliations:** ^1^Independent Researcher, Wellington, New Zealand; ^2^Department of Psychology, Liverpool Hope University, Liverpool, United Kingdom

**Keywords:** facial expressions of emotion, inversion effect, *Same/Different* task, response times, dual-process model, ex-Gaussian distribution, multidimensional scaling, cluster analysis

## Abstract

**Introduction:**

We used images of facial expressions (FEs) of emotion in a speeded *Same/Different* task to examine (i) distributional characteristics of response times (RTs) in relation to inter-stimulus similarity and (ii) the impact of inversion on FE processing.

**Methods:**

Stimuli were seven emotion prototypes, posed by one male and one female, and eight intermediate morphs. Image pairs (*N* = 225) were presented for 500 ms, upright or inverted, in a block design, each 100 times.

**Results:**

For both upright and inverted FEs, RTs were a non-monotonic function: median values were longest for stimulus pairs of intermediate similarity, decreasing for both more-dissimilar and more-similar pairs. RTs of “Same” and “Different” judgments followed ex-Gaussian distributions. The non-monotonicity is interpreted within a dual-process decision model framework as reflecting the infrequency of identical pairs, shifting the balance between the *Same* and *Different* processes. The effect of stimulus inversion was gauged by comparing RT-based multidimensional scaling solutions for the two presentation modes. Solutions for upright and inverted FEs showed little difference, with both displaying some evidence of categorical perception. The same features appeared in hierarchical clustering solutions.

**Discussion:**

This outcome replicates and reinforces the solutions derived from accuracy of “Different” responses reported in our earlier companion paper. We attribute this lack of inversion effect to the brief exposure time, allowing low-level visual processing to dominate *Same*/*Different* decisions while elevating early featural analysis, which is insensitive to face orientation but enables initial positive/negative valence categorization of FEs.

## Introduction

Using images of facial expressions (FEs) of emotion, we measured response times (RTs) while participants made “Same”–“Different” judgments on inter-pair similarity. FE pairs were presented both upright and inverted, in the hope that the thus-obtained RT measure would be a sensitive probe of the inversion effect. In the present report, submitted for the Research Topic “Methods and Applications in Perception Science,” we focus on RT distributions as functions of inter-stimulus similarity and the stimulus presentation condition. The aim is to analyze latency of stimulus discriminability, in its relation to an accuracy measure, to further explore processes of perceptual decisions underlying comparisons of visually complex stimuli.

The problem of determining whether two visual stimuli are identical is a natural activity with ecological implications. In experimental psychology, this function is operationalized as the forced-choice *Same/Different* (*S/D*) task, which has been widely used as a convenient psychometric technique for measuring (dis)similarities among a number of stimuli (for reviews, see Farell, 1985, 2022). The *S/D* task has been applied in a range of domains including schematic facial expressions (Takane and Sergent, 1983); line segments (Young, 1970); abstract symbols (Sergent and Takane, 1987); letters (Podgorny and Garner, 1979); irregular polygons (Cooper, 1976; Cooper and Podgorny, 1976; Smith et al., 2008); or single-syllable words (Farell, 2022).

In a *S/D* task, for *N* stimuli, the (*N*^2^ − *N*) pairs of different stimuli are each presented some number of times in random order, interspersed with repetitions of the *N* identical stimulus pairs. The latter provide no similarity information, but in their absence the observers could simply respond “Different” at every trial (though see Becker, 2012; Experiment 2). Non-identical pairs are recognized as such in the majority of trials if exposure times are long enough that inter-stimulus dissimilarities are above the threshold of discrimination. Indices of subjective dissimilarity are the average latency or response time (RT) required to decide that two stimuli differ and the proportion of correct “Different” responses to a given pair (i.e., accuracy).

Assuming that median RTs are a function of subjective dissimilarity, a preliminary to later analysis is to determine the nature of that function. Precedents for this postulate include several studies where RTs were related to inter-stimulus dissimilarities provided directly by subjects in the form of ratings (e.g., Young, 1970; Podgorny and Garner, 1979; Paramei and Cavonius, 1999).

In one widely-accepted form, this postulate states that for trials where different stimuli are correctly recognized as such, the median RT declines steadily as their dissimilarity increases, “an inverse monotonic function between the reaction time data and underlying distances” (Takane and Sergent, 1983, p. 396). This function slopes down steeply when the dissimilarity is subtle, i.e., a small increment in dissimilarity brings a large reduction in the difficulty of decisions, leveling out and approaching a floor value where the difference between the stimuli is immediately apparent (Cohen and Nosofsky, 2000). Following Shepard (1987), an exponential decline to a constant often fits the function well (e.g., Paramei and Cavonius, 1999).

Cooper (1976) reported that an exponential function fitted RTs from the majority of observers, although a minority appeared to apply a different decision process, and their RTs followed a flat function, not varying significantly with inter-stimulus dissimilarity (see also Cooper and Podgorny, 1976). Conversely, “just the opposite relation for [incorrect] “same” judgments was experimentally demonstrated [… implying] that for the “same” judgments, reaction time works as a measure of dissimilarity” (Takane and Sergent, 1983, p. 396). A preliminary objective here is to examine the universal truth of the assumption.

Another perspective looks at the entire *distribution* of RTs and responses for a given inter-stimulus dissimilarity, not just the measure of central tendency, and sets out to derive these from first principles (Balota and Yap, 2011). Of note are random-walk models (Laming, 1968), the diffusion model (Ratcliff, 1978, 1985), the race model (e.g., Huber and O’Reilly, 2003), and others, all falling under the rubric of dual-process decision models. These all agree in postulating two competing “evidence accumulators,” one receptive to any points of difference between the stimuli, and the other to the points on which they agree. These “accumulators” function in parallel until one or other function reaches a threshold. When the evidence for a “Same” decision outweighs that for a “Different” decision, or vice versa, then, depending on the metaphor of choice, the scales tip or the race is won. We will use “*Different*” and “*Same*” to label the decision processes, and “*S*” and “*D*” for the ultimate *response*.

Below, we examine the distributions of RTs for compatibility with dual-process decision models. Naturally this requires a large enough number of trials per stimulus pair (*T*). Larger values of *T* also make the average RT more robust, reducing noise from the many hard-to-control variables. There is a trade-off with observer motivation, however, not to mention the danger that observers will learn to recognize each pair as a single Gestalt and provide stereotyped, “over-learned” responses. In previous explorations of RTs as a function of similarity, *T* has ranged from four (e.g., Roberson et al., 1999, section 5.2), through 10 (Paramei and Cavonius, 1999), up to about 40 or 60 (Mollon and Cavonius, 1986). Much larger values are possible in studies attempting to model the underlying decision mechanisms, which typically examine fewer stimulus pairs. The present study used *T* = 100.

Another aspect of a *S/D* design is the proportion of identical stimulus pairs. The norm for studies in the *S/D* paradigm is to present equal numbers of identical- and different-stimulus trials (e.g., Qiu et al., 2017). Smith et al. (2008) reported that in a situation with about 50% of identical-pair trials, most human subjects followed a “zero-tolerance” decision strategy, responding *D* to any detectable disparity. In contrast, macaque monkeys appeared to impose a non-zero threshold, responding *S* or *D* to disparities below or above this threshold (as if comfortable with a high number of false-“Same” errors). This can be understood as the *Different* and *Same* processes of a decision model having separate thresholds to attain.

The thresholds can be manipulated by the experimental design: Ratcliff and Hacker (1981) influenced the RTs and relative numbers of *D* and *S* decisions by instructing observers to exercise greater caution before one response or the other. Downing (1970) influenced RTs by manipulating the proportion of trials where the stimuli were identical (50% vs. 25%). In Smith et al.’s (2008) experiment with humans, different-stimulus trials slightly predominated (54%) over same-stimulus pairs. As a precedent, in Wise and Cain’s (2000) study about 20% of pairs were identical. Following Krueger and Shapiro’s (1981, p. 576) reasoning, it is likely that variation in the ratio of same- and different-stimulus pairs (“heterogeneity of difference”) would shift the perceptual decision criterion. Specifically, they argued that decreasing the proportion of same-stimulus pairs reduces the amount of sensory evidence required to assign the *S* response, thus, resulting in greater number of false-“Same” errors. Indeed, when Smith et al. (2008) manipulated the ratio of identical- vs. different-stimulus pairs (30:35 vs. 40:35) in macaque monkeys, this induced a large shift in tolerance of stimulus disparity in a monkey presented with the 40:35 proportion of identical pairs, i.e., a looser, more inclusive criterion for responding “Same.” This finding is relevant to the present study due to the relatively low proportion of stimulus pairs (7%) which were identical.

In research on FEs of emotion, *S/D* accuracy data have been interpreted as dissimilarities and used to locate the category boundary between distinct emotions (Calder et al., 1996; Roberson et al., 1999; Suzuki et al., 2005). Such data are also suitable for multivariate analyses such as multidimensional scaling (MDS) and hierarchical cluster analysis, employed to reconstruct the perceptual framework underlying the stimuli, in order to glean clues as to (dynamics of) their cognitive representation.

The present study examined RTs for “Same” and “Different” judgments among images of FEs presented as pairs in upright and in inverted mode. Stimuli contained prototypical posed expressions of emotions and morphed intermediates. We estimated and scrutinized RT functions for individual subjects, while probing the effect of stimulus inversion upon the encoding and processing of FEs in terms of proximities among them in a spatial model. In particular, we asked whether the inversion impacts more upon some emotions than others; and whether, after inversion, emotion categories still modulate the perception of FEs. A previous MDS analysis of response accuracy in the same experiment (Bimler et al., 2013) found unexpectedly little effect from inversion, and one question we examine here is whether the RTs, as a complementary behavioral measure of (dis)similarity, reveal more effect when examined with the present approach.

## Materials and methods

### Participants

Two male and two female undergraduate Psychology students, aged 21–25 years, were reimbursed for participation. All participants were right-handed and reported normal vision. Participant sex and poser gender were counterbalanced to offset any possible own-gender bias effect in face recognition (*cf.* Wright and Sladden, 2003). That is, stimuli from the MO series (from a female poser) were presented to one female participant (DK) and one male (HK). Likewise, the WF series (from a male poser) was presented to one female participant (SB) and one male (BF). Each participant completed 30 1-h-long sessions spread over 4 months; for HK and SB these were interrupted by a three-month gap during their summer vacation. The study was conducted in accordance with the ethical principles of the Declaration of Helsinki.

### Stimuli

Fourteen grayscale photographs of emotional expressions were selected from *Pictures of facial affect* (Ekman and Friesen, 1976). Those authors deemed these 14 images to be good examples of seven universal emotion categories in unalloyed form [*Happiness (H), Surprise (Su), Anger (A), Sadness (Sa), Fear (F), Disgust (D), Neutral (N)*], as evinced by high accuracy of labeling. Seven images featured a female poser identified as MO while the other featured a male poser WF. (see Figure 1 in Bimler et al., 2013).

**Figure 1:**
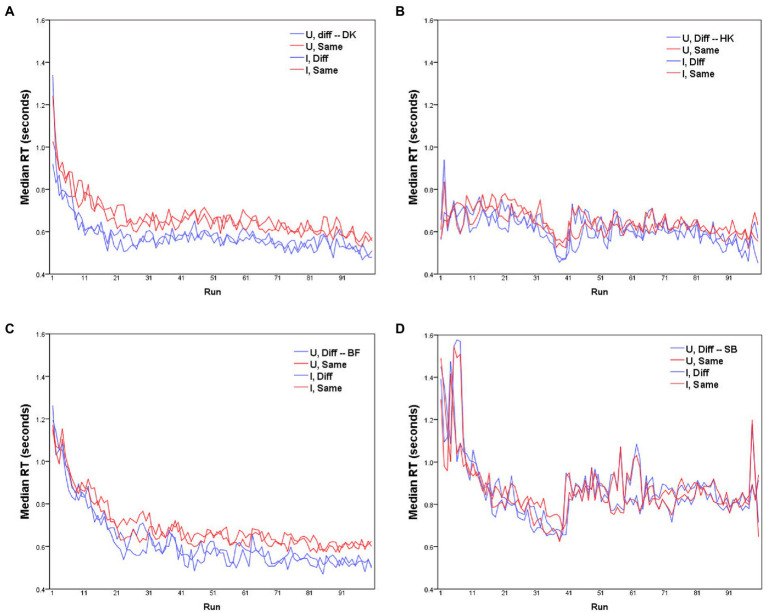
Median RTs for each of the four participants **(A–D)** as a function of run, for *D* responses (blue lines) and *S* responses (red lines), with Upright and Inverted stimuli (solid and dashed lines respectively). MO series: participants **(A)** DK, **(B)** HK; WF series: participants **(C)** BF, **(D)** SB.

The MO series and WF series were both extended by using image-interpolation software (Design Studio) to create eight ambiguous intermediate stimuli, each lying midway along the continuum defined by two emotion exemplars as end-points. The morphing process involves ‘landmarks’ located within each prototype, allowing smooth interpolation of intermediate stages along a transformation between them (*cf.* Calder et al., 1996; Young et al., 1997). Using morphs between all 21 (7 × 6/2) pairs of “parent” exemplars would have made data collection impractical, so eight pairs were chosen, following a distorted circumplex that paired each exemplar with its neighbor (e.g., *SaN, DF*), except for *Anger*, which is paired with *Happiness* (*AH*) and *Surprise* (*ASu*). Each set, MO and WF, included 15 images.

These digitalized stimuli were presented on a 19” CRT-Monitor (V7 N110s), where each image occupied 12.8 cm × 8.7 cm (subtending 10° × 6.7° at a viewing distance of 74 cm). Measured with a LMT L1009 Luminance Meter, image luminance ranged from 0.23 to 82 cd/m^2^. Ambient lighting in the test room was in the mesopic range (around 10 cd/m^2^).

### Procedure

Each trial consisted of the simultaneous parafoveal presentation of two FE stimuli, symmetrically side-by-side on the screen with a 3.8 cm gap between them (subtending 3°). After 500 ms, the screen went blank until the participant responded “Same” (*S*) or “Different” (*D*) via a two-button keyboard. Instructions described the stimuli as “emotional faces,” to focus the participants’ attention on their emotional content. Participants were instructed to respond as quickly and correctly as possible. RT was measured (to the nearest 20 ms) from the appearance of the FE pair to the response, by a MS-DOS program running on a Windows-98 PC which controlled presentation and recorded each *S* or *D* response. Each response was followed by an inter-stimulus interval of 300–400 ms, while a small red fixation cross was displayed on the monitor.

In a single run, all possible 15 × 15 = 225 pairings of FEs were presented in randomized order. Blocked presentation was used, alternating between blocks of all Upright (U) or all Inverted (I) pairs. For each participant the experiment began with a practice session of one block in each of the U and I modes. There followed 10 sessions containing six blocks and 20 containing seven blocks, totalling to 100 runs with FE pairs in the U mode and 100 runs in the I mode.

Note that 15 of these 225 pairs were indeed identical (7%). The remaining pairs consisted of “left–right” and “right–left” presentations of 105 pairings. These were treated as repetitions in the analysis, ignoring any asymmetry effects. Participants received no indication about how frequently to expect identical pairs, and no feedback about accuracy after trials.

## Results

### Learning effect

Before further analysis, the data require some rescaling to compensate for any learning effect. Median RTs in each run are plotted for the four participants separately in Figure 1. Clearly these values change across the course of data collection, with some participants showing substantially shorter RTs with accumulated practice (for HK and SB, the abrupt increase in RTs after the 40th trial reflects their summer interruption).

**Figure 2:**
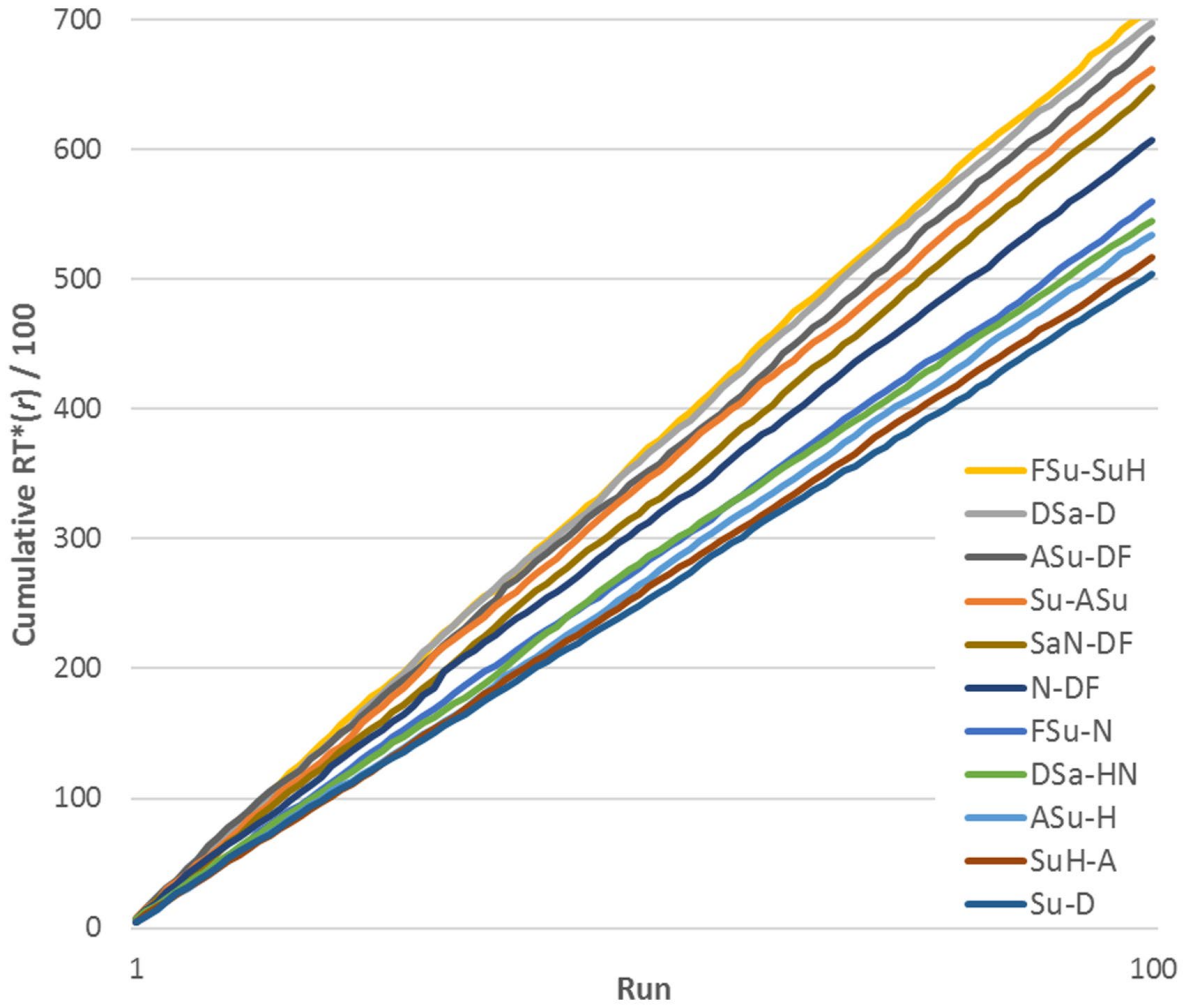
Cumulative sum of RT_ij_*(*r*) across 100 runs for 11 representative stimulus pairs (exemplified by data for DK, Upright stimuli).

A natural concern is the possibility that RTs for a given pair varied systematically in the course of the experiment relative to other pairs, as subjects became familiar with the stimuli. To test this, we plotted *cumulative sums* of RT_ij_* for representative stimulus pairs, across a range of similarities (see Figure 2, exemplified by subject DK, Upright pairs). RT_ij_* is defined in the next paragraph. The lines are reasonably straight and do not cross, implying that RTs remained quite stable relative to the median at each run. Thus, Figure 2 shows that changes from learning were across-the-board and did not change the *relationships* among pairs: if a pair evoked a relatively rapid response in the initial runs, it was still relatively rapid at the end of the experiment. However, this progressive change increased the variance of the distribution of RTs for a given pair of FEs.

### RTs as a measure of FE inter-stimulus similarity

Following Ratcliff et al. (2010), median RTs for each FE pair were obtained separately for *D* and *S* responses, $M_{ij}^{D}$ and $M_{ij}^{S}$ . U and I mode trials were analyzed separately. The *median* value is preferable to the mean, being unaffected by the skewed distribution of RTs or the outlying, exceptionally delayed responses that sometimes occur. As noted, participants’ response speed varied as the runs progressed, typically improving with practice. To remove this source of variance, before calculating their medians the 225 RTs in each run *r* (1 ≤ *r* ≤ 100) were rescaled with a factor *s*(*r*) to bring their median value into line with the global median over all runs for that participant:


$$RT*ij\left(r\right)=s\left(r\right)\mathrm{RTij}\left(r\right)$$

where *s*(*r*) = global median(RT*_ij_*)/median(RT*_ij_*(*r*)). We also performed the same analyses without this adjustment, but found no impact on the overall tenor of the outcomes.

Errors occurred relatively often with observers responding *S* in about 25% of the trials (*cf.* 7% of actually identical pairs). The percentage of erroneous *D* judgments for a given pair served as a proxy for the perceived dissimilarity between those stimuli, and was constant across runs. In a companion paper we processed accuracy rate (percentages) of *S* judgments as an index of pairwise perceptual similarity with non-metric multidimensional scaling (MDS) for error-smoothing purposes, embedding them within a four-dimensional geometrical space (Bimler et al., 2013).

### RTs to identical FE pairs

First, we explored RTs to identical pairs of FEs, separately for Upright and Inverted conditions. The (objectively) identical pairs of the MO series stimuli most rapidly identified as “Same” were *H-H*, *FSu-FSu*, and *Su-Su*, while the slowest pairs were *N-N*, *SaN-SaN*, *ASu-ASu* and *Sa-Sa*. For the WF series, the pairs with the shortest “Same” RTs were *H-H*, *F-F*, and *A-A*, while the slowest pairs were *N-N* and *DF-DF*. These outcomes concur with Becker’s (2012) report that in a *S*/*D* task, matched pairs of negative images took longer to recognize as identical than neutral or positive-affect pairs, a finding attributed to greater demands of processing negative expressions. However, that the most rapidly processed stimuli are marked not so much by their positive affect, but rather by the clarity of a single feature (e.g., WF’s exaggerated smile for *Happiness*, or MO’s elevated eyebrows and open mouth for *Surprise*). That is, the results are consistent with the observers noticing that the stimuli of a pair share a specific exaggerated feature, apparently tipping the scales toward a *S* response and curtailing further thought. This finding is also in accord with Calvo and Nummenmaa’s (2011) conclusion that early (and later) expression discrimination decisions are based on visual saliency of distinctive facial features.

### RTs of “Different” vs. “Same” responses to FE pairs

**Figure 3:**
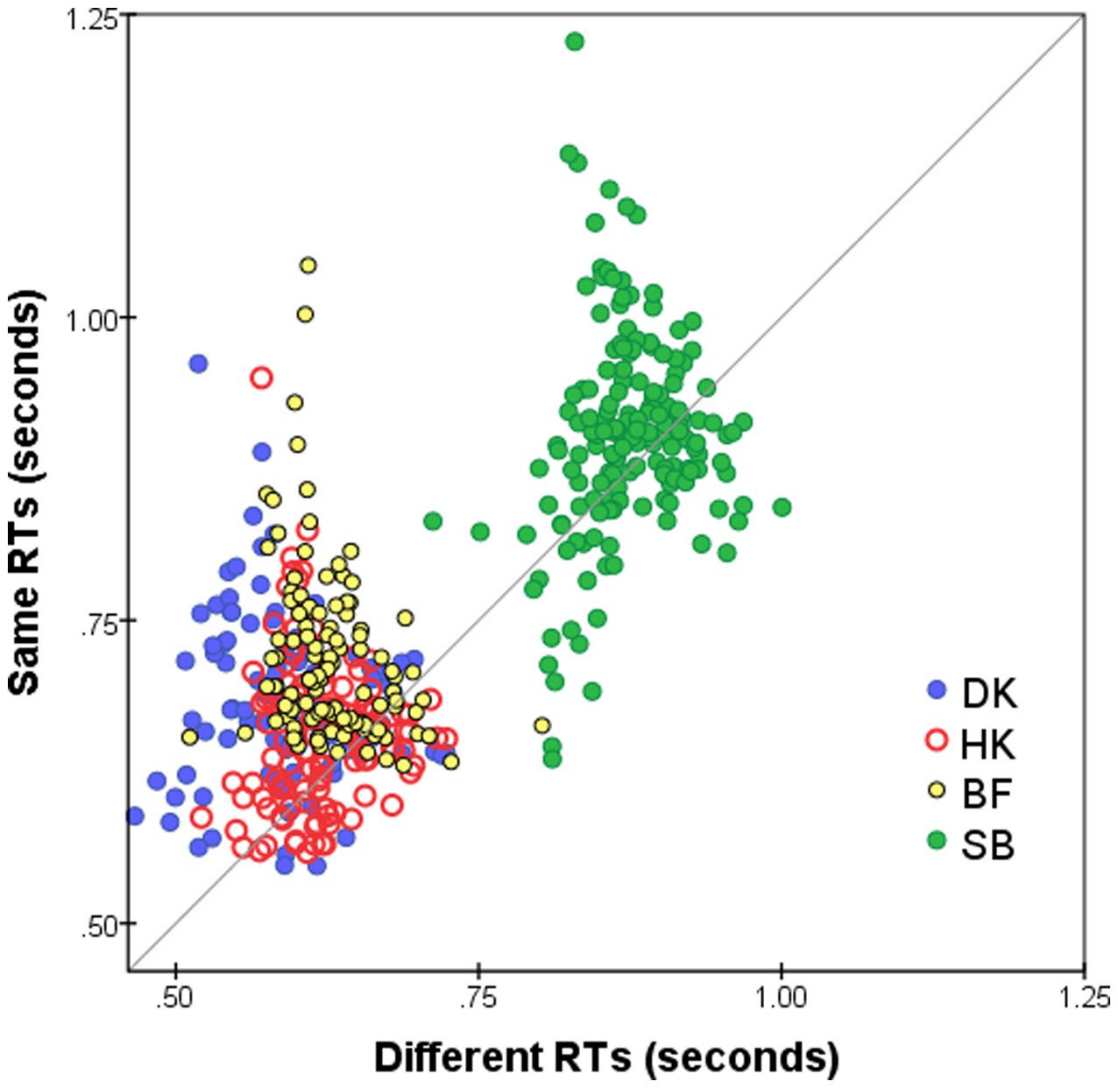
Median RT* $M_{ij}^{S}$ for *S* responses to *i*-th and *j*-th FEs, *i* ≠ *j* (vertical axis), plotted against $M_{ij}^{D}$ for *D* responses (horizontal axis). Superimposed results for four observers and for both Upright and Inverted presentation modes. Pairs omitted if four or fewer responses.

Median RTs for *S* responses ( $M_{ij}^{S}$ ) were slightly longer than for *D* responses ( $M_{ij}^{D}$ ), as is evident in Figure 3 that plots $M_{ij}^{D}$ on the horizontal axis against $M_{ij}^{S}$ for the same pairs on the vertical axis. The slower *S* responses indicate a more conservative decision criterion, in accord with previous findings: *S* responses (conjunctive judgments) imply accumulating more evidence before making the decision whereas for *D* responses (disjunctive judgments) a decision is made as soon as any difference is detected (see Farell, 1985, for a review). The delay in the *S* responses—mean( $M_{ij}^{S}$ − $M_{ij}^{D}$ )—is not constant for all stimulus pairs but varies as a function of pairwise dissimilarity. In addition, mean( $M_{ij}^{S}$ − $M_{ij}^{D}$ ) varies from subject to subject, with the largest average delay for BF and DK (90 and 95 ms, respectively) and least for SB (27 ms). It is possible that these inter-individual differences in the delay of *S* responses are spurious, since in RTs of HK (Figure 1B) and SB (Figure 1D) there was an abrupt increase after the summer interruption (we are indebted to a reviewer for this caveat).

### RT distributions: Dual-process model

Considering RTs from the dual-process perspective, their distributions become relevant to the possible effects of inversion. In the dual-process paradigm, one can imagine the response to a given stimulus pair (*i,j*) as a Bernoulli model, where the visual system reports to the *Same* and *Different* processes at a regular rate (i.e., at regular clock ticks), and each report has a constant chance of being *Same* and *Different*, this chance depending on how many features the stimuli share (*cf.* Ratcliff, 1978). The decision process continues until the *Same* or *Different* detector has accumulated enough reports to trigger either a *S* or *D* response, respectively.

**Figure 4:**
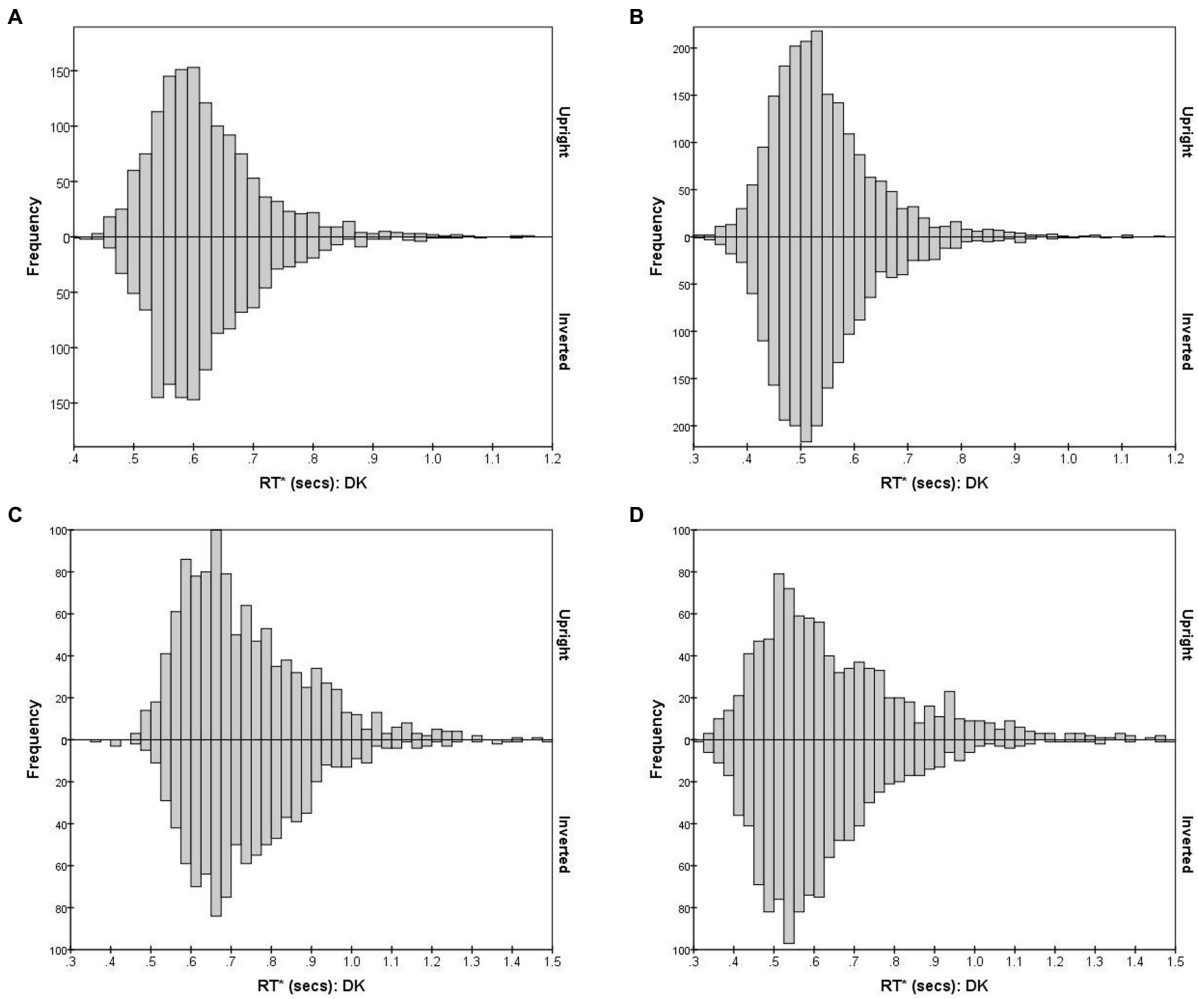
RT*(*i,j*) distributions for observer DK, for FE pairs presented Upright (positive values on the *y*-axis) and Inverted (negative values on the *y*-axis). The four graphs illustrate RT* (*i,j*) distributions for pairs that vary in the degree of inter-stimulus similarity. **(A)** *S* responses for 15 identical pairs; **(B)** *D* responses for 10 most-different pairs; **(C)** *S* responses for 10 intermediate-similarity pairs; **(D)** *D* responses for 10 intermediate-similarity pairs.

Figure 4, using observer DK as example, shows the combined distribution of RT*(*i,i*) for FE pairs varying in subjective (dis)similarity, separately for the Upright and Inverted mode. (Results for the other observers are shown in Supplementary Figure S1.) Figure 4A shows the distribution of RT*(*i,i*) for the 15 identical-stimulus pairs. These pairs (*i,i*) evoke the *Same* process without interruption from *D* responses and the *S* threshold is almost always reached. Figure 4B shows the combined RT*(*i,j*) distributions for 10 pairs which were most distant in DK’s accuracy-based MDS solution. Conversely, the *Different* process manifests in isolation in this case of pairs of greatest dissimilarity, where the *D* threshold is almost always reached (RTs for the 15 identical pairs were pooled here, as were the 10 most-dissimilar pairs, to reduce statistical noise in the histograms.) The Bernoulli model predicts *S* and *D* RTs to follow negative binomial distributions, positively skewed, if the accumulation of reports is uninterrupted. As predicted, for DK (and also for the other observers; see Supplementary Figure S1), both distributions are positively skewed with a long “tail” of delayed RTs.

The situation is more complicated for pairs of intermediate dissimilarity. Figures 4C, D plot the RT*(*i,j*) distributions for erroneous *S* and correct *D* responses, combining 10 pairs (*i,j*) lying within a band of intermediate distances, chosen so that errors were closest to 50% of responses, i.e., these were pairs for which the dual-process competition was seemingly strongest.

When the reports from the visual system have equal probability of being *S* and *D*, the *Same* and *Different* functions accumulate at only half the rate as in the extreme cases, predicting longer-delayed and therefore less skewed negative binomial distributions. But an additional factor is at play. The probability of a *S* conclusion after some time *t* is reduced by the cumulative probability that a *D* response had already emerged at any time < *t* (so the theoretical *S* distribution is modulated by the *cumulative* distribution for the *Different* process). Conversely, the distribution of *D* responses for these pairs is shaped by the cumulative distribution of the *Same* process. Details of this two-way interaction depend on the relative speed of the two processes, among other factors, which might make these specific distributions most sensitive to any effects of inversion.

**Figure 5:**
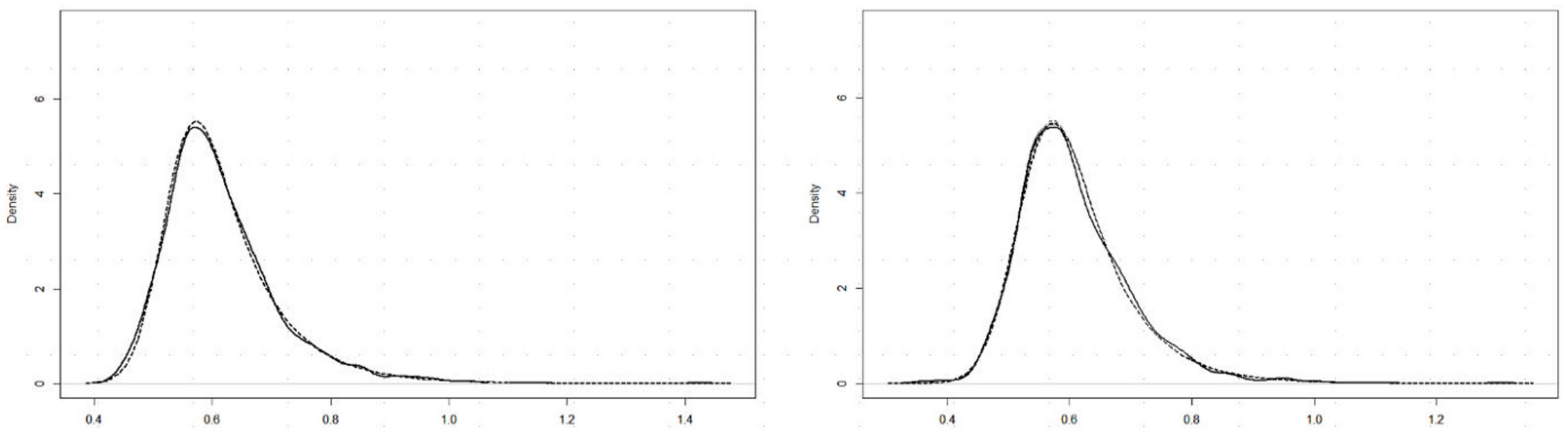
RT*(*i*,*j*) distributions for 15 identical pairs (solid lines) with superimposed ex-Gaussian functions (dotted lines) for observer DK, and Upright (left) and Inverted (right) presentation mode.

Past RT data have been successfully modeled by an exponential-Gaussian function with parameters μ, σ, τ (e.g., Heathcote et al., 1991, 2019; Balota and Spieler, 1999). Accordingly, we applied the “timefit” function from the “retimes” (package for R), to *S* response RT*(*i,j*) values to identical pairs (exemplified by data for participant DK, U mode), as in Figure 4A. The matches between the resulting ex-Gaussian functions and actual distributions for this participant are gratifyingly close (Figure 5), validating the data transformation and suggesting that median values are valid measures of central tendency. Table 1 shows the function parameters and the corresponding moments (mean, standard deviation, skewness) for all observers. (Density functions for the other three observers are presented in Supplementary Figure S2.) Note that in line with previous findings (e.g., Heathcote et al., 1991; Balota and Spieler, 1999), the ex-Gaussian characteristics of individual participants are relatively stable regardless of the mode of FE presentation.

**Table 1:** Parameters (μ, σ, τ) for fitted ex-Gaussian functions and moments (mean, SD, and skewness) for the distributions of *S* RTs for identical FE pairs in Upright and Inverted mode of presentation, for each observer (Ob).

Mode	Upright						Inverted					
Obs	μ	σ	τ	Mean	SD	Skew	μ	σ	τ	Mean	SD	Skew
DK	0.528	0.043	0.086	0.614	0.096	1.439	0.529	0.048	0.078	0.607	0.092	1.250
HK	0.519	0.046	0.080	0.598	0.092	1.291	0.522	0.043	0.090	0.611	0.099	1.461
BF	0.560	0.044	0.061	0.621	0.075	1.070	0.565	0.046	0.054	0.619	0.070	0.886
SB	0.736	0.067	0.058	0.794	0.089	0.558	0.735	0.056	0.067	0.802	0.087	0.910

### Non-monotonicity of the RT function

**Figure 6:**
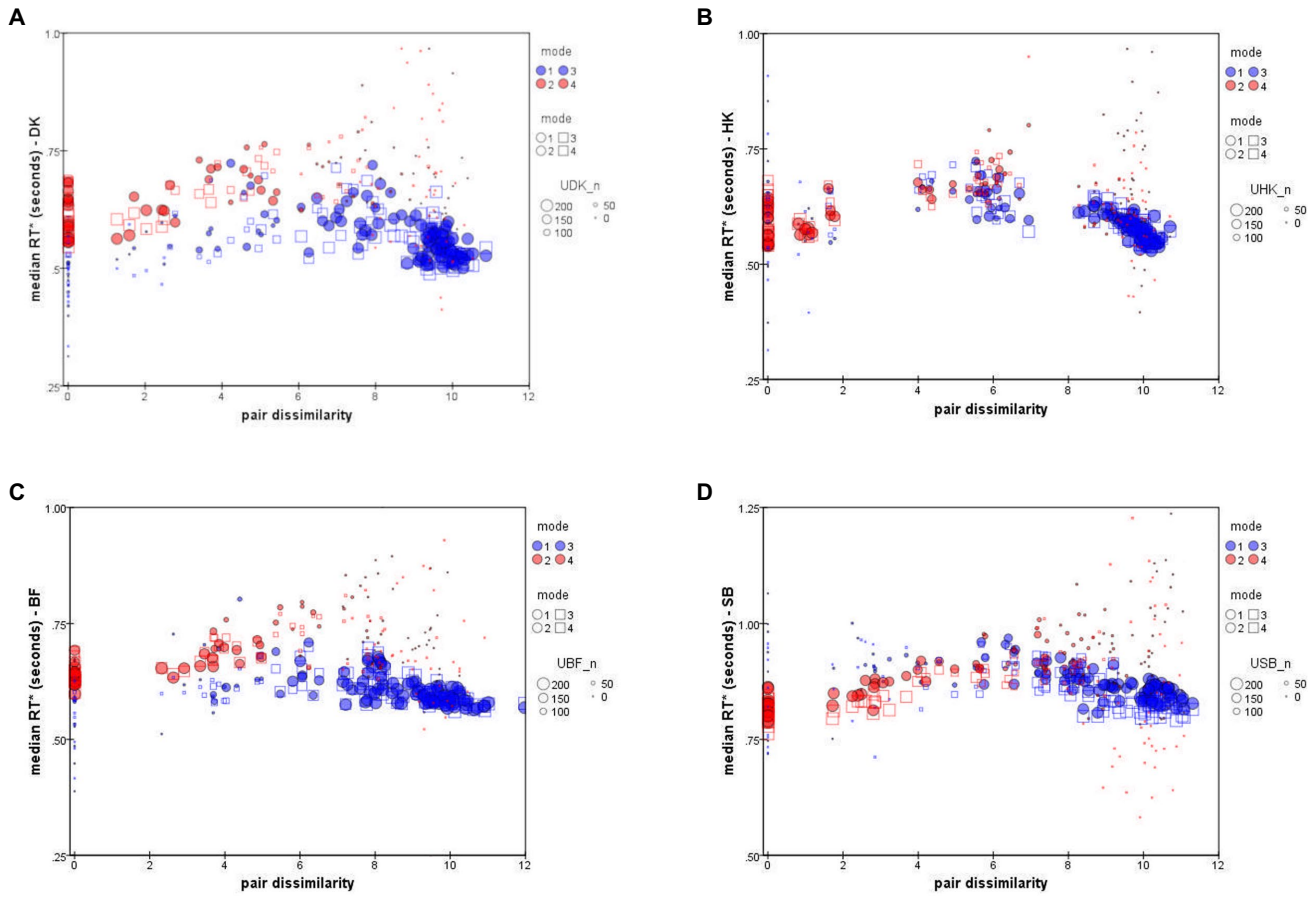
Median RT* of “Same” responses, $M_{ij}^{S}$ (red symbols), and “Different” responses, $M_{ij}^{D}$ (blue symbols), as a function of distance in the accuracy-rate based MDS solutions. ● = Upright mode; □ = Inverted mode. Symbol size represents *number* of responses to pair (*i,j*). Data for individual participants: **(A)** DK, **(B)** HK, **(C)** BF, **(D)** SB (note difference in the *y*-axis scale).

Figure 6 plots each observer’s median RTs $M_{ij}^{D}$ and $M_{ij}^{S}$ against inter-stimulus dissimilarity (i.e., inter-point distance in that subject’s accuracy-based MDS solution; *cf.* Bimler et al., 2013). To indicate the reliability of data-points, the size of each symbol represents the number of decisions on which that median is based.

The unexpected feature is that the $M_{ij}^{D}$ values follow a peaked function rather than an exponential decline or any other monotonic function (see also Paramei et al., 2009). This is particularly clear in the results for HK, Figure 6B, who took longest to make a *D* response for an intermediate dissimilarity of about *dist* = 6 (arbitrary units in the MDS solution). As expected, more-distant pairs were judged in less time, but so were more-similar pairs.

The other three observers exhibit comparable non-monotonicity for $M_{ij}^{D}$ . The $M_{ij}^{S}$ distributions also follow a non-monotonic contour, though with slightly larger values, the relative delay varying from subject to subject. The shape is less clear because there were very few *S* responses for highly-dissimilar pairs, so that *S* points at the right of each panel of Figure 6 are based on only a few atypical responses, limiting their reliability.

We note that SB consistently took 300 ms longer to respond than the other participants (Figure 6D), although the *pattern* of her RTs is no different. Until further observers are tested, we do not know whether SB is anomalous or at the conservative end of a range of processing-criteria variation, causing a more exhaustive, attention-strengthened comparison strategy with greater cognitive control (*cf.* Moret-Tatay et al., 2016).

### Filtering RT data prior to calculate MDS solutions

For further analysis we applied MDS to estimates of similarity derived from median RTs. The solution represents stimuli as points in the spatial model, where the proximity of any two points mirrors the corresponding stimulus similarity, and dimensions indicate attributes underlying the perceptual judgments.

To compensate for the non-monotonicity of $M_{ij}^{D}$ as functions of reconstructed distances, we filtered their values to the range where they were monotonic, by abandoning all entries for stimulus pairs (*i*,*j*) that were similar enough for fewer than 33% of trials to return *D* responses (the exact threshold is not crucial). The result is a *similarity* matrix SIM_D for each observer and each presentation mode, where the matrix elements are *sim_d_ij_*:


$$\begin{array}{c} sim\_d_{ij}=MD_{ij}\;\mathrm{if fraction of}\;D\;\mathrm{responses}<33\%\\ =\left[\mathrm{missing}\;\mathrm{data}\right]\;\mathrm{otherwise} \end{array}$$

The effect is to retain only pairs from the *right-hand* side of each panel of Figure 6, i.e., the ones that contain information about (sufficiently) large dissimilarities which determine the global structure of MDS solutions. To provide complementary evidence about the finer structure among adjacent stimuli, a second matrix DISS_S was included in the same analysis, consisting of $M_{ij}^{S}$ values treated as *dissimilarities*—but only for those stimulus pairs where the $M_{ij}^{D}$ value was rejected, with [*missing data*] entries otherwise (That is, the entries of this second matrices came from the monotonic *left-hand* half of each $M_{ij}^{S}$ vs. distance function shown in Figure 6).


$$\begin{array}{c} \mathrm{dissim}\_s_{ij}=MS_{ij}\;\mathrm{if fraction of}\;S\;\mathrm{responses}>66\%\\ =\left[\mathrm{missing}\;\mathrm{data}\right]\;\mathrm{otherwise} \end{array}$$

It follows from the filtering rule that if a stimulus pair (*i*,*j*) is represented by its $M_{ij}^{D}$ value in a filtered SIM_D matrix, while another pair (*k,l*) is omitted there but is represented by its $M_{\mathrm{kl}}^{S}$ value in the corresponding DISS_S matrix, then (*k,l*) is more similar than (*i,j*). We emphasize that the MDS analysis below does *not* use this inference in any way.

### RT-derived MDS solutions

**Figure 7:**
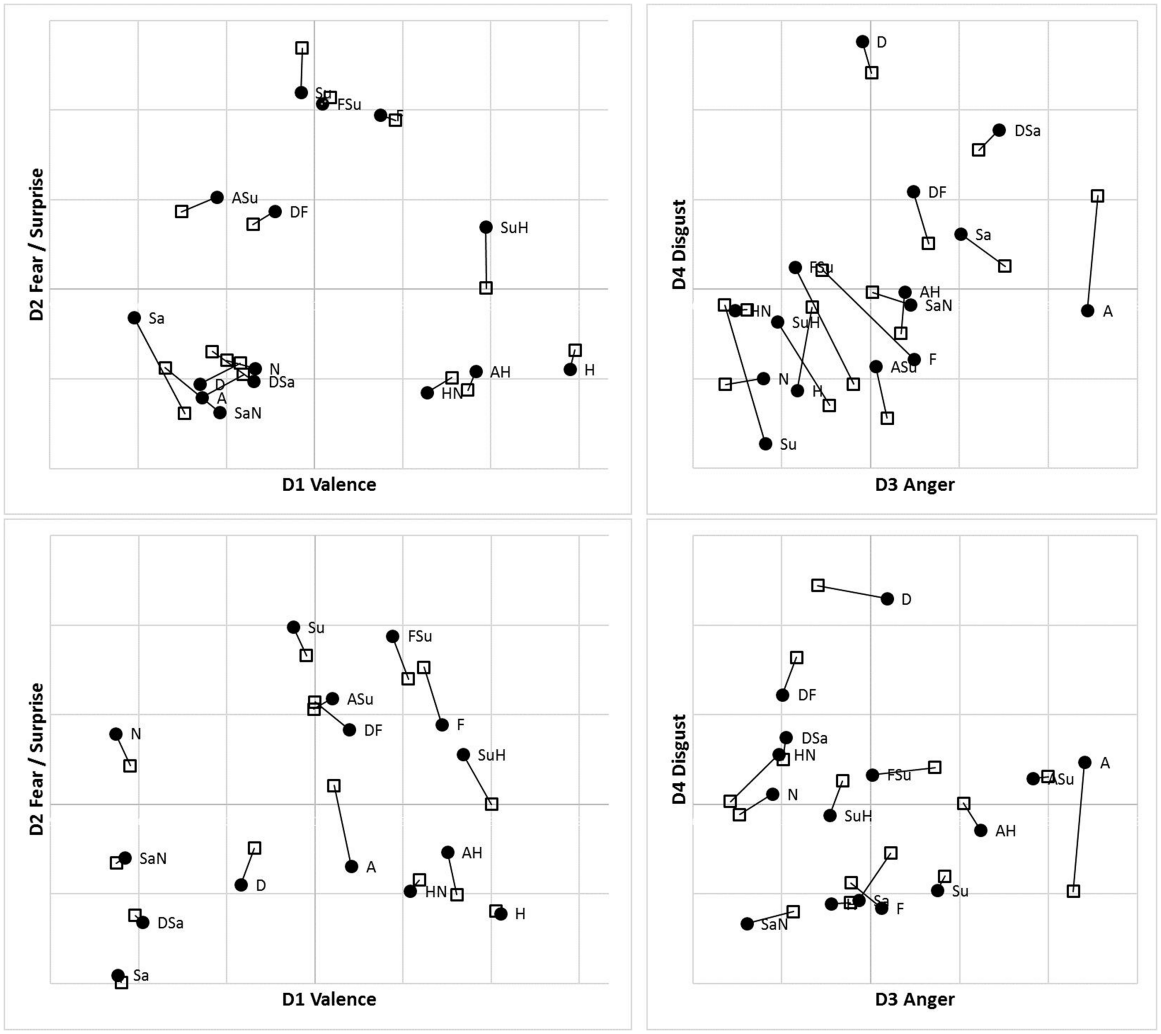
Four-dimensional MDS solutions for MO (top) and WF (bottom) stimulus sets, from “filtered” RT*(*i*,*j*) medians *sim_d_ij_* and *diss_d_ij_*, pooling two observers for each stimulus set and superimposing solutions for Upright (●) and Inverted (□) observation (linked by lines). Projection on D1D2 (left) and D3D4 (right) planes.

Following accuracy-based analysis in Bimler et al. (2013), we retained four-dimensional MDS solutions for the MO and WF series separately, for each presentation mode, using an implementation of Kruskal’s algorithm in its multiple-matrix repeated-measures mode to pool two subjects’ SIM_D and two DISS_S matrices for each poser. Notably, and unexpectedly, no systematic inversion-related differences appeared between solutions for the U and I data, so they are superimposed in Figure 7 as two sets of points, using Procrustes analysis (Gower, 1975) to rotate each pair of solutions to the closest congruence. Given this similarity, we pooled the U and I data to obtain consensus MO and WF solutions (not shown). Values of *Stress*_1_ for the 2D to 4D solutions were 0.144, 0.105, 0.088 (MO) and 0.178, 0.126, 0.101 (WF). These *Stress*_1_ values and interpretability of all four dimensions justify retention of four dimensions in both cases. After rotation, as expected, the first dimension D1 is a bipolar “Valence” axis, distinguishing the *Happiness* stimulus and its morphs at one extreme from negative-valence FEs at the other. The other axes are unipolar, running from “Neutral” to “Fear/Surprise” (D2), “Anger” (D3) and “Disgust” (D4; see Figure 7).

For confirmation we derived a dissimilarity function (*d_ij_*), and created dissimilarity matrices **D**^MO^ and **D**^WF^, by defining the difference between the *i*-th and *j*-th stimulus in terms of the *profiles* of median RTs involving them (the respective rows $M_{ik}^{D}$ and $M_{jk}^{D}$ in the similarity matrix). Specifically, *d_ij_* is the Euclidean distance between rows $M_{ik}^{D}$ and $M_{jk}^{D}$ :


$$d_{ij}=\sqrt{\sum_{k}}{\left(MD_{ik}-MD_{jk}\right)}^{2}$$

wherei≠j≠k .

Note that in contrast with the original RT data, this dissimilarity function *d_ij_ is* a monotonic function of the reconstructed distances. Four-dimensional MDS solutions for the MO and WF series (each pooling the matrices for two participants and the two presentation modes) had *Stress*_1_ of 0.134 and 0.141, respectively, and were encouragingly similar to those obtained above.

### Comparison of solutions derived from RTs and accuracy rates

These combined RT-derived solutions were compared to the solutions extracted from the accuracy-rate data (Bimler et al., 2013). Similarity between MDS solutions derived from the two behavioral measures was quantified in several ways. One is the Procrustes statistic *R*^2^, measuring the total sum of residual distances between corresponding points that remain when the configurations have been rescaled, translated, reflected and rotated so as to maximize the overlap between them (Gower, 1975). A value of *R*^2^ = 0 indicates complete convergence of the two structures. In this case the values were small: *R*^2^ = 0.051 when comparing the solutions from accuracy rates and RTs for the MO series, and *R*^2^ = 0.032 for the WF series.

A second form of comparison, canonical correlation (CANCORR), has the advantage of allowing significance tests in the form of Wilks’ Λ statistic, here a very stringent test with only 15 points for the correlations. For both the MO and WF series, all four dimensions of the RT solution have recognizable counterparts in the accuracy-rate solution, with *p* ≤ 0.002 and *p* < 0.005, respectively.

### Effect of FE inversion

**Figure 8:**
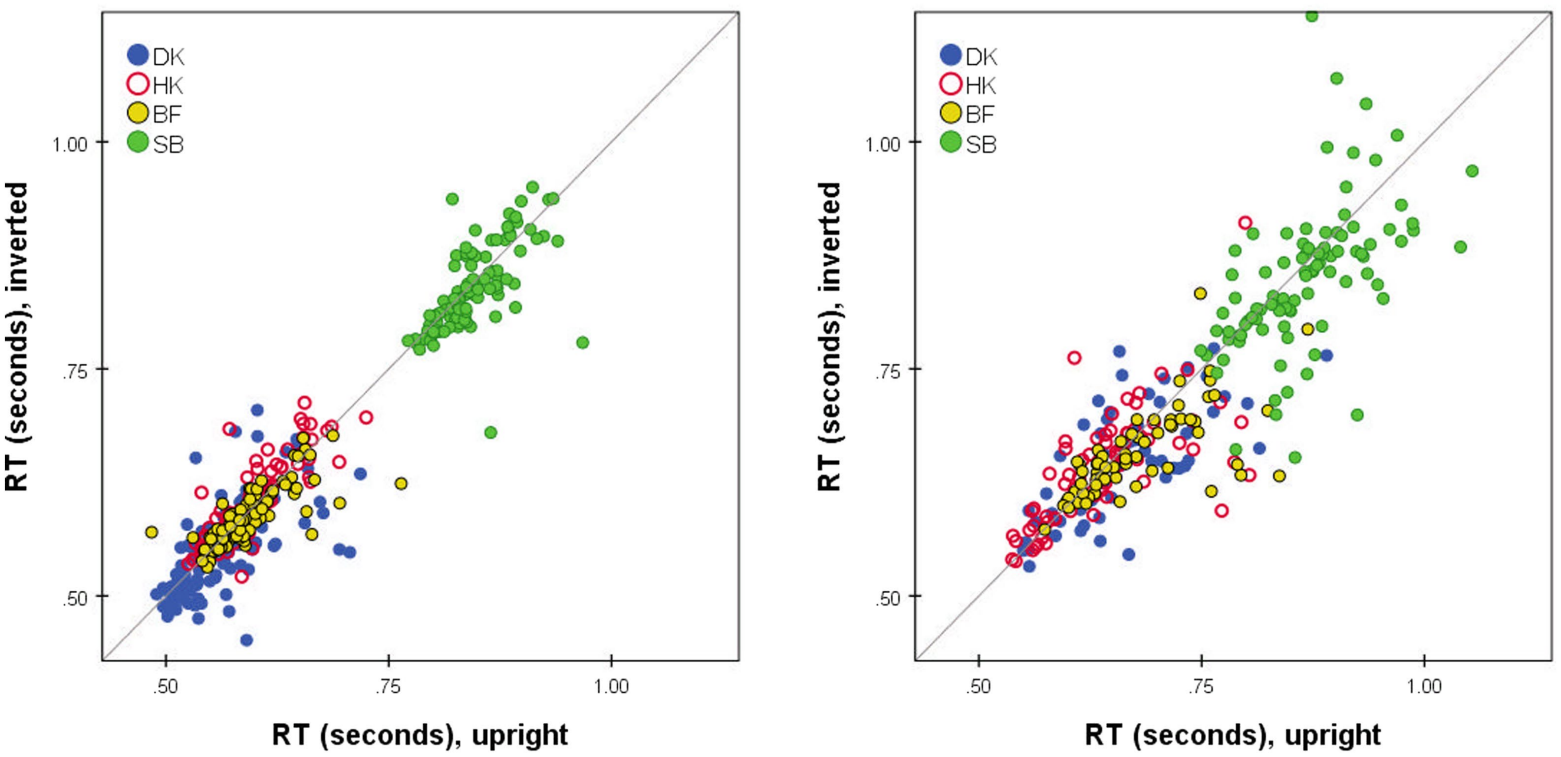
Median RT*(*i*,*j*) for Upright pairs (horizontal axis) vs. Inverted pairs (vertical axis). $M_{ij}^{D}$ for *D* responses (left); $M_{ij}^{S}$ for *S* responses (right). Superimposed results for four observers. Pairs omitted if four or fewer responses in either presentation mode.

No glaring difference between the RTs to Upright and Inverted pairs of stimuli was apparent (Figures 1, 4, 6, 7). At a finer level of analysis, Figure 8(left) plots the *D*-response median RTs $M_{ij}^{D}$ for each pair of Inverted stimuli against $M_{ij}^{D}$ for the identical pair when presented Upright. In the same way Figure 8(right) plots the *S*-response median RTs $M_{ij}^{S}$ . It is apparent that inversion failed to substantially affect processing time: the points are concentrated around the diagonal. Significant variations are in the minority (before correcting for multiple comparisons). Median *D* RTs of participant HK were shorter for Upright pairs than for Inverted pairs by 4.8 ms, *p* = 0.029; in comparison, SB gave faster *D* and *S* responses to Inverted than Upright stimuli, by 21.9 and 40.9 ms, respectively, both *p* < 0.001. Crucially, these unsystematic within-participant differences were far smaller than differences between the observers, with DK as the fastest responder and SB as the slowest.

**Figure 9:**
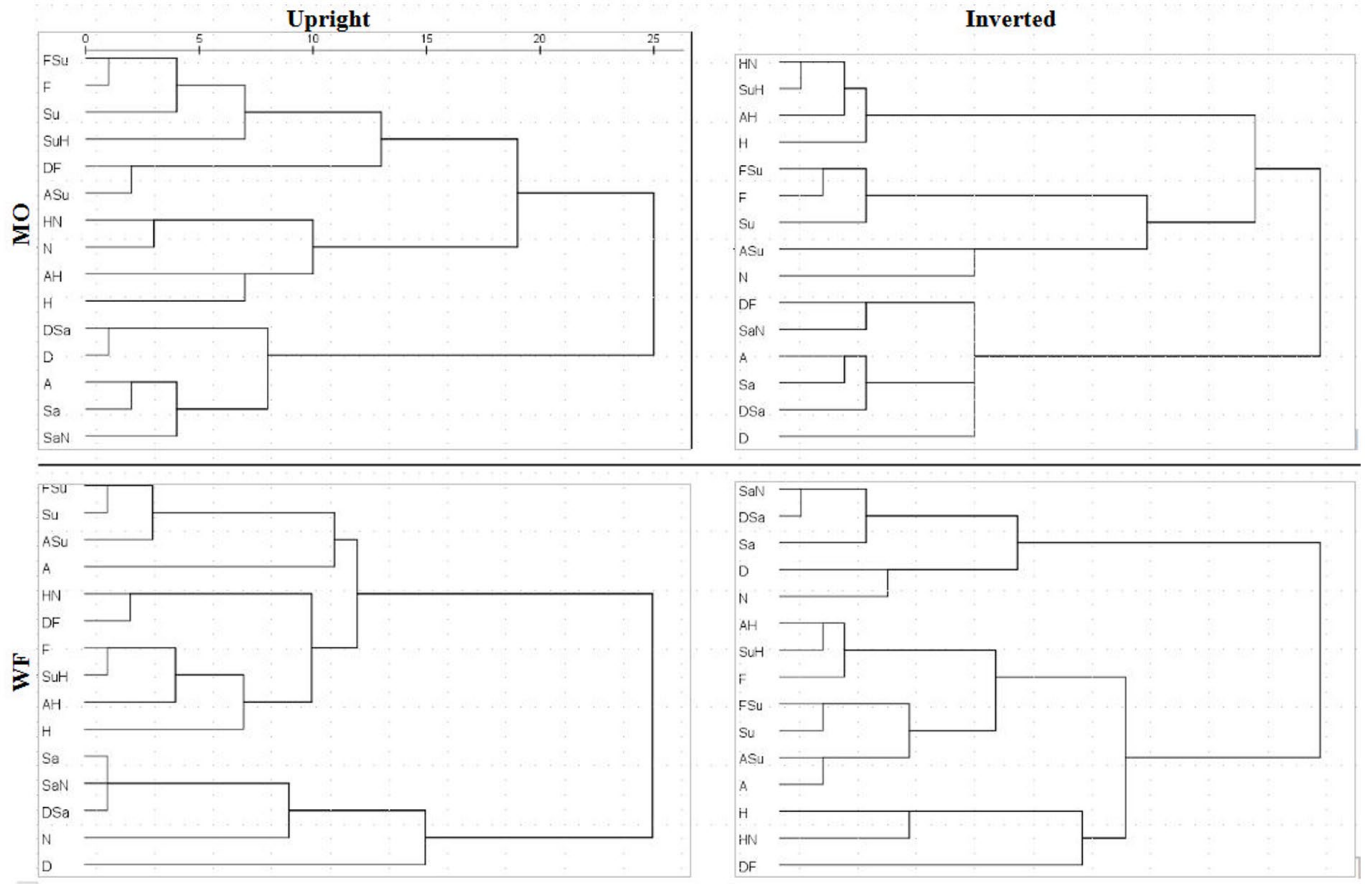
Dendrograms derived from MDS solutions for median RT*(*i*,*j*) for MO series (upper row) and WF series (lower row), Upright (left) and Inverted (right).

A detailed comparison of 4D structures is difficult when working with 2D perspectives, where a high-dimension rotation can shift points’ locations in unexpected ways. Conversely, apparent clustering of points may be coincidental overlaps. To facilitate comparison of the MDS summaries of filtered RTs, we processed the distances within each solution with hierarchical clustering analysis (HCA), specifically, mean link agglomerative algorithm. HCA results (dendrograms) for Upright and Inverted images of the MO and WF series are shown in Figure 9. HCA cannot be applied to the SIM_D and DISS_S matrices directly due to their missing-data entries.

If the subjective similarities represented in MDS solutions strictly follow the construction of the stimulus set, one expects clusters in the HCA that contain a pair of the prototype FEs and their intermediate morph. For instance, taking *Sadness* and *Neutral* expressions as a relatively similar pair of prototypes, where the *SaN* stimulus is (objectively) between at an equal distance from both, if *SaN* clusters with *Sadness*, then it should be equally similar to *Neutral*, drawing the latter into the cluster. The same might occur for *Fear*, *Surprise* and *FSu*.

For the MO stimuli, as expected, both U and I solutions exhibit a cluster of *Fear/Surprise/FSu*, part of a high-level three-way division (*ASu* is a peripheral member of the cluster, but not the *Anger* prototype). Further, the “negative” emotions *Sadness*, *Anger* and *Disgust* and their morphs comprise a second cluster, joined by *SaN* but not *Neutral*, contrary to the equal-similarity assumption. Finally, the three half-*Happiness* morphs *HN, SuH* and *AH* have coalesced with the *Happiness* prototype into the third cluster. That is, the morphs were all more similar to *Happiness* than to their other “parent” prototypes: the response times appear to reflect a degree of categorical processing, which persists when stimuli are inverted. It is worth noting that the same features are present in hierarchical-clustering solutions for accuracy-rate similarity matrices (not shown).

We observed, however, that inversion *does* shift some morphs from the periphery of one cluster to another. In particular, *SuH* and *DF* are both in the *Fear/Surprise* group according to Upright responses, but inversion shifts *SuH* to the *Happiness* cluster and *DF* to the “negative” cluster. Conversely, *Neutral* is in the *Happiness* cluster according to Upright responses (linked by its proximity to *HN*), but is in the *Fear/Surprise* group when inverted, where the connection is less obvious. One cannot read too much into these details, as the cluster membership of ambiguous stimuli is susceptible to random fluctuations in the data.

The WF solutions, in comparison, exhibit a two-way split, distinguishing a cluster of *Sadness*, *Disgust*, *Neutral* and their morphs. Other stimuli are more of a continuum, affected by data fluctuations. The *Happiness* prototype and morphs do not coalesce completely for Inverted data, but *Fear*, *AH* and *SuH* do form a consistent tight cluster. Another tight cluster can be discerned in both U and I results, combining *Surprise* with *FSu* and *ASu*, along with *Anger* (drawn by proximity to *ASu*) but not *Fear*. Recall that these local details are driven by the DISS_S matrices, in contrast to the SIM_D matrices determining the global structure.

## Discussion

### Learning effect

Following the outline of the Results section, we begin by addressing possible learning effect on RTs. The unusually large number of trials and responses in this study (*T* = 100 per stimulus pair) highlight certain important aspects of RTs. First, they vary with practice—generally becoming shorter, albeit with relapses. This will not surprise anyone who has played computer games or learned to touch-type, engraining automatic motor pathways by dint of time and practice. Importantly though, these fluctuations were not accompanied by any systematic shift in accuracy rates across the course of data collection, as assessed by the discrimination *d’* and bias *C* parameters (Bimler et al., 2013). It would seem that the improvements are limited to the motor skill of pressing one or the other key. Crucially for present purposes, RTs for specific pairs show no progressive changes over runs when rescaled to be proportional to the median RT for a given run.

This learning effect is not a serious concern here because the drift affects the median RT to the same extent across stimulus pairs. However, extensive practice does increase the *variance* around each median, obscuring the shape of the distributions, which provides the rationale for reducing that variance with the corrections applied here. Presumably similar practice shifts also occurred, although to a lesser extent, in RT data gathered with fewer *S/D* trials (e.g., Takane and Sergent, 1983).

### Individual variation

The variations among observers are of note. Even within the small group studied here, SB responded more slowly than the others with a smaller relative advantage for *D* responses (Figures 1D, 3), and a relative advantage for Inverted pairs (Figure 8). It may be that personality traits influence these differences in decision and, hence, dual-process parameters. For example, Nowicki and Cooley (1990) reported that subjects with internal locus of control were significantly faster to distinguish different emotions in a *S/D* task. Cognitive style may, also, underlie differences in an individual’s strategy of attention allocation, reflected by an eye-movement pattern of exploration of the compared images that affects the process and chronometry of a perceptual decision (for a discussion *cf.* Bendall et al., 2016).

The ex-Gaussian functions fitted to the RT data (Figure 5; Table 1) may be relevant here. The μ and σ parameters of the normal component characterize the “leading edge” of a distribution and are conjectured to reflect early automatic processing, whereas τ, the exponential component characterizing the length of the tail, is more likely to reflect central attentional process (e.g., Balota and Spieler, 1999). As seen in Table 1, μ and σ are higher for SB than for other participants in both U and I modes while τ is lower, i.e., distribution skewness. Moret-Tatay et al. (2016) attribute lower τ to reducing either the tendency to double-check by a participant before responding or the amount of attentional lapsing. One can speculatively interpret SB’s lower τ as fewer attentional lapses than other participants’, i.e., her “attentional-based strengthening” due to “enhancement in cognitive control” (in the authors’ terms) was higher, also increasing μ and σ. However, as noted by Heathcote et al. (1991) and Matzke and Wagenmakers (2009), the rationale for ex-Gaussian functions is more empirical than theoretical, so we have not attempted to interpret the parameters further.

### The role of the ratio of same- and different-stimulus pairs in the design

Another factor that possibly affected the obtained outcome is the imbalance of (factually) same- and different-stimulus pairs in our experimental design. The identical-stimulus pairs in the present study, 7%, were scarce compared to the default design in RT research of 50% of total trials. This aspect of the design, facilitated by the brief presentation time, might have encouraged observers to overlook points of difference (or to pay more attention to points of similarity), biasing their judgments toward erroneous *S* responses (*cf.* Krueger and Shapiro, 1981). It may be that in the absence of explicit guidance for how many “same” pairs to expect, and in the absence of corrective feedback, participants implicitly set their own targets for what seemed a plausible rate and adjusted the thresholds of their decision processes accordingly. In the dual-process framework, the present design is likely to have had an impact on the drift rate of information accrual in the dual processes (*cf.* Ratcliff, 1978) and, hence, the balance between them. More specifically, it seems to have elevated the critical level of stimulus disparity required for an *S* or *D* judgment to be equally likely (with observers accepting disparities below this criterion as ‘sufficiently identical’), a strategy regarded by Smith et al. (2008) as characteristic of non-human primates. The predominance of different-stimulus pairs appears to have the unintended effect of increasing the number of erroneous *S* responses, with the benefit of enhancing the statistical robustness of false *S* rates when they serve as an index of stimulus similarity. As a result, we were previously able to use each subject’s percentage of *D* responses to each stimulus pair as a yardstick of “dissimilarity” (Bimler et al., 2013).

We also observed that *S* judgments were made more slowly than *D* judgments for the same-stimulus pair (Figure 3), although the delay varies with pair and with the participant. This may be another outcome of the change of processing criteria, in accordance with Downing’s (1970) report that *S* responses were slower when they were less frequent (25% vs. 50% of trials). Future studies could examine the effects of variation in the proportion of same- and different-stimulus pairs on shifting the criterion of perceptual decisions, potentially reflected by accuracy rate and mean RT difference of *S* and *D* responses.

### The RT function of stimulus similarity does not sustain monotonicity

The outcome of the observers’ relaxed criterion of “sameness” in the present study revealed a feature of the *Same/Different* task that normally is obscured. In particular, the data become a test of the assumption that RTs are a monotonic function of stimulus dissimilarity, assessed by accuracy (“%*D*”; Bimler et al., 2013) or by distances in a RT-derived MDS solution as a smoothed version, as here. As demonstrated in the Results section, in either case, despite the simplicity and intuitive appeal of the assumption, it fails for the present data.

We argue, with Ratcliff (1985) and also Ratcliff et al. (2010), that this non-monotonic RT/dissimilarity relationship is in fact an inherent feature of the task, usually concealed but brought to the foreground by aspects of the present study. When the experimental design allows a non-identical stimulus pair to attract a substantial number of *S* responses, it becomes possible for the *Same* process to forestall the *Different* process, and thereby truncate the distribution of RTs for *D* responses. For relatively similar pairs, the *S* responses become the majority, and they are also brief (because their underlying RT function is *decreasing* with similarity). Thus, *D* responses are recorded only on the trials when the *Different* process happens to handle the visual information even more rapidly—probably because some unmistakable, visually salient point of difference between the stimuli “popped out” during their presentation.

Non-monotonicity is a natural corollary of a dual-process decision model. We argue that it is not observed in studies where there are 50% of same-stimulus pairs with zero disparity which attract the majority of *S* responses, these being correct. In such cases the response threshold for the *Same* process arguably is higher than in the present data (or the threshold for the *D* response is lower). This shifts the cross-over between the two processes—the level of dissimilarity where either response to a pair is equally likely, and the *D*-response distribution is truncated—to a difference too small to appear in any non-identical stimulus pairs.

If, as proposed above, the observers here have adjusted their decision criteria and handicapped the *Different* process, as it were—shifting their thresholds to increase the fraction of *S* responses—this would produce Figure 6 as a side-effect. Note that Podgorny and Garner (1979), who reported RTs for *D* responses to same-stimulus pairs, indeed found that, consistent with our prediction, these incorrect *D* responses were actually *shorter* than RTs for many different-stimulus pairs, presumably because of the *Same* process forestalling longer responses. The principle with which we began—that “reducing dissimilarity increases the median value of the distribution of *Different* RTs”—is only true in the special case that the dual processes of the decision model can operate in isolation. This account predicts that RTs for *D* responses will peak at the dissimilarity for which the two kinds of responses are equally common, for that observer. Inspection of the data shows this to be the case.

The same argument further predicts that median RTs of *S* responses will also be a non-monotonic function of dissimilarity, because at larger dissimilarities where the *Different* process operates rapidly we only see the truncated lower tail of the *S* distribution, from those trials where the *Same* process has operated more rapidly still. As noted, this was the case for DK. Such responses are rare, however. There is an additional complication that a *S* response to a dissimilar pair can also occur if the observer was unable to respond promptly (due, e.g., to inattention during the 500 ms of presentation), leaving neither process with adequate information beyond a fading memory trace, and forcing a delayed and effectively random response. Consistent with this hypothesis, here RTs were generally long when subjects gave *S* responses for dissimilar pairs (often exceeding 1 s). Such “timed-out” trials in this scenario can also yield long *D* responses, but they are lost in the large majority of rapid *D* responses, having little impact on $M_{ij}^{D}$ .

### MDS solution derived from a corrected RT function

When the non-monotonicity is recognized it can be corrected, making the data suitable for MDS. Several lines of evidence converge to validate the RT-derived MDS solutions obtained here. In particular, these solutions concur with geometric models for the same stimuli obtained previously by interpreting the accuracy rate as a dissimilarity measure (Bimler et al., 2013). Furthermore, all present RT-derived solutions are plausible with regard to both formal and explanatory MDS criteria: they have low values of badness-of-fit (*Stress_1_*); their dimensions lend themselves to straightforward interpretation as continuous affective gradients; and they are internally consistent as models of the relationships among FE stimuli (specifically, the point representing each morph is located somewhere between the points for the prototype FE “parents”). This validates the use of median RTs as a measure of perceptual difference (in particular, for probing category effects) in the more common situation where the error rate is *not* high enough to serve as an index of proximity. Here we concur with Wise and Cain (2000, p. 261), who summed *S/D* errors across subjects and across bands of stimulus pairs, to support their conclusion that “latency to discriminate shows promise as an objective measure of qualitative similarity.”

### No effect of inversion on discrimination of facial expressions

An unexpected result was that inversion of stimulus pairs had no consistent effect on response times (Figures 1, 7) or the relative order of RTs (Figures 6, 8), despite our expectation that the emotional content of inverted FEs would require slower serial analysis of local features due to the disruption of processing configural cues, whereby distal features are integrated into a unified whole (Rossion, 2008). One obvious explanation is that, in the challenging discrimination task, the affective content was simply not involved in the *S/D* decisions, these being made purely on the basis of similarity of face trivial details or visually salient diagnostic features (Arnold and Lipp, 2011; Calvo and Nummenmaa, 2011; Murphy et al., 2020; Baldassi et al., 2022), and increase in contrast (in the mouth region for happiness and the eye region for fear; Psalta and Andrews, 2014) or of perceived *physical* similarity, with the stimuli undergoing a low-level form of comparison as abstract patterns of gray-tones and textures (*cf.* Bimler et al., 2013).

However, there is some evidence of an effect of inversion upon RTs and accuracy rates, but this is confined to pairs of intermediate dissimilarity where the competition between the dual processes is strongest, and thus susceptible to small changes in their parameters. Comparing the Upright and Inverted RT*(*i*,*j*) mode distributions for intermediate-similarity pairs for DK indicates that this observer identified more of the sufficiently-similar pairs as *D* when Inverted (Figure 4D) and (erroneously) as *S* when upright (Figure 4C). Responses of HK followed the same pattern, with inversion increasing the median *D* RT (Supplementary Figure S1). However, this subtle difference is not consistent across observers, and for SB the effect of inversion was to increase the number of correct *D* responses (Supplementary Figure S1, third row), in addition to allowing faster responses than in Upright presentation (Figure 8). These pairs were outnumbered by pairs that were sufficiently similar or sufficiently dissimilar for the *Same* or *Different* process to operate without interference, and they were lost in the MDS solutions.

### Feature-based early extraction of FE affective meaning

The weakness of any FE inversion effect is in accord with previous findings in two studies measuring response speed—one using a visual search paradigm (Lipp et al., 2009) and another on the choice of a face with the highest emotional content in a horizontally aligned pair (Baldassi et al., 2022), where both suggest a role for low-level processing of face images. Even so, the present study demonstrates partial extraction of affective information despite the brief stimulus presentation. Specifically, the “Valence” dimension is extracted early in visual processing of facial expressions, in line with previous studies using visual search (Lipp et al., 2009) or forced-choice paradigms (for a review see Calvo and Nummenmaa, 2016). As argued by Calvo and Nummenmaa (2011, p. 1758), it is possible that affective information is extracted at some point but is “only minimally *used* due to [it] being overshadowed by the earlier extracted, and simpler to be managed, visual saliency information, which then was retained also for later discrimination stages.” This conclusion is in accord with findings in an ERP study demonstrating that an emotional expression effect is recorded as early as 120–180 ms post-stimulus (Eimer and Holmes, 2007).

The exposure of 500 ms may have been too short for the processing of the configural information involved in the full decoding of affective meaning (*cf.* Rossion, 2014; Calvo and Nummenmaa, 2016; Murphy et al., 2020) but it was evidently long enough to extract low-level visual cues at early stages of processing face expressions, shown to be manifested in ERPs within 200 ms (Batty and Taylor, 2003; Ashley et al., 2004; Ohmann et al., 2016; Duan et al., 2022) or relatively correct affective estimates of FEs presented for 150 ms (Arnold and Lipp, 2011). A further corollary can provisionally be drawn from the Results, that some sufficiently low-level facial property distinguished *Sadness*, while no such property was unique to *Fear* or *Surprise* (any low-level cues being shared between those last two expressions).

### Early *Happiness* categorical perception effect

Further, along with the subjective dichotomy of the expression “Valence,” our results reveal an early categorical perception (CP) effect for *Happiness*-images (cf. Bimler and Kirkland, 2001). Although the nature of the stimulus set precludes a rigorous test of CP, the RT-based dissimilarities do display certain hallmarks of CP. In particular, some of the interpolated morphed *Happiness*-stimuli subjectively are not midway between their two constituent prototypes, but are displaced toward one (i.e., are harder to distinguish from it). The *Anger-Happiness*, *Surprise-Happiness* and *Happiness-Neutral* morphs (*AH, SuH*, and *HN*) were all closer to the *Happiness* prototype than to their other constituent, although the *HN* morph (for instance) is physically just as close to *Neutral*. This would be the outcome if they fell within the boundary of the *Happiness* category. Notably, this structure of the subset of *Happiness*-stimuli is present in separate MDS solutions, congruent with our previously-reported accuracy-based outcomes of this study (Bimler et al., 2013). The emerged *Happiness-*subsets in the RT-derived MDS solutions are in accord with outcomes of cluster analysis (see Figure 9 in the present paper). In addition, it is buttressed by Fechner analysis outcomes of our accuracy data: *K*-means clustering revealed a *H, AH, SuH*, and *HN* cluster, for both U and I conditions and all participants (Dzhafarov and Paramei, 2010). The early emerging *Happiness* category concurs with the finding of the N170 component that reflects earliest manifestation of the CP effect (Qiu et al., 2017; Duan et al., 2022).

The *Happiness* categorization effect is also visible in the hierarchical clustering extracted from Upright and Inverted RTs (Figure 9). The *AH*, *SuH*, and *HN* morphs combine with the *Happiness* prototype in a distinct cluster: in speeded similarity decisions, the 50% of *Happiness* dominates the 50% of other emotional prototypes, regardless of inversion. This early manifestation of the *Happiness* category concurs with both the timing of psychophysiological components—a shorter latency of the N170 (Batty and Taylor, 2003) and categorization advantage (lower discriminability) of positive expressions (Qiu et al., 2017); and, as well, of behavioral measures of responses to *Happy* faces—shorter visual search times (Lipp et al., 2009), saccade latencies (Calvo and Nummenmaa, 2011; Beaudry et al., 2014), and faster choices of the “happiest” than the “angriest” face in a pair (Baldassi et al., 2022).

Our finding is also in accordance with the visual salience of a “smile” feature estimated using behavioral measures: Smith and Schyns (2009) and Bombari et al. (2013) found the *Happiness* expression to be low-spatial-frequency rich, involving the mouth as its distinguishing feature more than the other prototype FEs. Lower perceptual thresholds for *Happiness* detection compared with *Fear*, *Anger* or *Sadness* point out to the lip-end raise as a highly diagnostic feature (Calvo and Nummenmaa, 2011; Du and Martinez, 2013; Maher et al., 2014; Calvo et al., 2018). This distinctive single cue bypasses integration of face parts (*cf.* Calvo et al., 2014) and, in the present study, renders the categorical processing of briefly glimpsed facial *Happiness*, in both its “pure” and morphed forms, less susceptible to inversion.

## Concluding remarks

The absence of difference in the RT pattern of “Same”–“Different” judgments between Upright and Inverted FE pairs suggests that the participants were viewing the (briefly presented) faces not as Gestalts but, rather, as abstract patterns of low-level features (maybe as gray-tone gradients). In the above discussion of potential information-processing mechanisms behind the present findings we favored the explanation that leans upon the dual-process model and implies separate thresholds in the *Same* and *Different* accruing processes.

This explanation, though, is not the only possibility. We are grateful to an anonymous reviewer, who pointed out that this finding of no inversion effect implies that the *S/D* task is a search paradigm, whereby independent (low-level) stimulus components undergo an analytic and self-terminating comparison, a “feature search” or search of feature conjunctions (Farell, 1985, 2022). Our results (Figure 3) fit into a serial search paradigm with expected shorter *D* responses compared to *S* responses. However, as pointed out by Farell (1985) in his seminal work, the relation between the pattern of “Same”–“Different” judgments and parallel vs. serial processing is difficult to determine.

## Data availability statement

The raw data supporting the conclusions of this article will be made available by the authors, without undue reservation.

## Ethics statement

The studies involving human participants were reviewed and approved by Institute of Psychology, Darmstadt University of Technology, Germany. The participants provided their written informed consent to participate in this study.

## Author contributions

DB conducted the data analysis and contributed to writing the manuscript. GP contributed to the study design, running the experiment, initial data processing, and writing the manuscript. All authors contributed to the article and approved the submitted version.

## Conflict of interest

The authors declare that the research was conducted in the absence of any commercial or financial relationships that could be construed as a potential conflict of interest.

## Publisher’s note

All claims expressed in this article are solely those of the authors and do not necessarily represent those of their affiliated organizations, or those of the publisher, the editors and the reviewers. Any product that may be evaluated in this article, or claim that may be made by its manufacturer, is not guaranteed or endorsed by the publisher.

## Supplementary material

SUPPLEMENTARY FIGURE S1 RT* distributions, Upright and Inverted, for HK (top row), BF (middle row) and SB (bottom row). (**a**) *S* responses for 15 identical pairs; (**b**) *D* responses for 10 most-different pairs; (**c**) *S* responses for 10 intermediate-similarity pairs; (**d**) *D* responses for 10 intermediate-similarity pairs.

SUPPLEMENTARY FIGURE S2 RT*(*i,j*) distributions for 15 identical pairs (solid lines) with superimposed ex-Gaussian functions (dotted lines) for three remaining participants (rows), in Upright (left) and Inverted (right) modes of FE presentation.
